# A randomized trial of desflurane or sevoflurane on postoperative quality of recovery after knee arthroscopy

**DOI:** 10.1371/journal.pone.0220733

**Published:** 2019-08-05

**Authors:** Stuart Boggett, Jared Ou-Young, Johan Heiberg, Richard De Steiger, Martin Richardson, Zelda Williams, Colin Royse

**Affiliations:** 1 Department of Surgery, The University of Melbourne, Melbourne, Victoria, Australia; 2 Dept. of Cardiothoracic & Vascular Surgery, Aarhus University, Aarhaus, Denmark; 3 Epworth Healthcare Campus, The University of Melbourne, Melbourne, Victoria, Australia; 4 Department of Anaesthesia and Pain Management, The Royal Melbourne Hospital, Melbourne, Australia; 5 Outcomes Research Consortium, Cleveland Clinic, Ohio, United States of America; Geneva University Hospitals, SWITZERLAND

## Abstract

**Background:**

Studies have described different recovery profiles of sevoflurane and desflurane typically early after surgery.

**Methods:**

We conducted a randomized superiority trial to determine whether Overall Recovery 3 days after knee arthroscopy would be superior with desflurane. Adult participants undergoing knee arthroscopic surgery with general anesthesia were randomized to either desflurane or sevoflurane general anesthesia. Intraoperative and postoperative drugs and analgesics were administered at the discretion of the anesthesiologist. Postoperative quality of recovery was assessed using the “Postoperative Quality of Recovery Scale”. The primary outcome was Overall Recovery 3 days after surgery and secondary outcomes were individual recovery domains at 15 minutes, 40 minutes, 1 day, 3 days, 1 month, and 3 months. Patients and researchers were blinded.

**Results:**

300 patients were randomized to sevoflurane or desflurane (age 51.7±14.1 vs. 47.3±13.5 years; duration of anesthesia 24.9±11.1 vs. 23.3±8.3 minutes). The proportion achieving baseline or better scores in all domains increased over the follow-up period in both groups but was not different at day 3 (sevoflurane 43% vs. desflurane 37%, *p* = 0.314). Similarly, rates of recovery increased over time in the five subdomains, with no differences between groups for physiological, *p* = 0.222; nociceptive, *p* = 0.391; emotive, *p* = 0.30; Activities-of-daily-living, *p* = 0.593; and cognitive recovery, *p* = 0.877.

**Conclusion:**

No significant difference in the quality of recovery scale could be shown using sevoflurane or desflurane general anesthesia after knee arthroscopy in adult participants.

## Introduction

Knee arthroscopy is a commonly performed outpatient surgical procedure. Inhalational anesthetics are often used as they enable rapid emergence from anesthesia with minimal postoperative side effects.[1]

The most frequently used inhalational anesthetics are sevoflurane and desflurane, which differ in their pharmacokinetic properties and hence, could have different effects on postoperative quality of recovery.[2–8] Comparative studies have shown that desflurane is superior to sevoflurane in terms of early recovery parameters such as return of pharyngeal reflexes and wakefulness,[2–4, 6–8] and also, it has been demonstrated that sevoflurane has considerable variability in these physiological parameters, leading to less predictable recovery times than desflurane.[2, 3] Conversely, the perioperative incidence of coughing has been found higher with desflurane than with sevoflurane.[6] Although several studies have described different recovery profiles of sevoflurane and desflurane, physiological recovery parameters have typically been the main focus.

Although the pharmacokinetic properties of the two volatile anesthetics are well described, there are few data on the longer-term effects on recovery, especially cognition. Previous data has mostly been restricted to pre-clinical animal studies. Our group has previously published on longer term cognitive function in aged rats exposed to anesthesia without surgery and have found considerable differences between volatile anesthetics, indicating that we should not consider that all volatile anesthetics will produce similar recovery profiles, though our animal studies have not shown major effects on cognition for sevoflurane and desflurane, but have shown long term cognitive effects for isoflurane. [9–12]. However, there is a lack of clinical effectiveness research examining cognitive changes and other recovery domains in patients in the time-period beyond pharmacokinetic elimination of the anesthetics. In patients undergoing longer and more complex surgery, differences in cognitive recovery have been shown days after surgery favouring desflurane against propofol TIVA for cardiac surgery [13] and hip replacement surgery (late difference not early). [14] For comparisons of sevoflurane and desflurane, Mahmoud et al [15] demonstrated a higher proportion of patients receiving desflurane returning to normal activity after brief gynaecological surgery. White et al [6] studied a heterogenous cohort of outpatient surgery patients and demonstrated that 60% of participants receiving desflurane resumed normal activities 1 day after surgery compared to 48% receiving sevoflurane, though this difference was not significant with the small sample size. Taken together, anesthesiologists should not assume that different anesthestics will produce the same recovery outcomes even after brief surgery. These data do not inform clinicians for a common clinical question—whether the two most commonly used volatiles, sevoflurane and desflurane, influence quality of recovery after brief surgery. A further issue with assessment of cognitive recovery in the early time period is that many patients will receive strong analgesics, which may confound the ability of any cognitive assessment battery to discriminate the effect on cognition from the anesthetic drug versus pain and strong analgesics. If cognitive effects of the anesthetic persist well beyond pharmacological elimination, then this difference may only be apparent after the requirement for strong analgesics has diminished. Three days after outpatient arthroscopy the requirement for strong analgesics should be minimal.

The Postoperative Quality of Recovery Scale (PostopQRS) is a multidimensional scale which includes a cognitive domain.[16] The PostopQRS differs from other scales as it is objective, designed for multiple timepoints and includes a cognitive domain [17], making it an ideal tool to investigate our clinical question. This assessment tool is able to collect data in an efficient and systematic manner whilst obtaining a rich cross-section of data in a standardized manner being able to identify where improvements in outcomes are needed.

In this study, the aim was to identify whether general anesthesia with either sevoflurane or desflurane affects the overall postoperative quality of recovery after knee arthroscopy, using the Postoperative Quality of Recovery Scale over multiple time points to 3 months after surgery. Secondary aims were to determine the effects of the two volatiles on other recovery domains—physiological, nociceptive, emotional, activity-of-daily-living, and cognitive recovery up to 3 months after surgery. The hypothesis was that there would be no difference between the volatile anesthetics on Overall Recovery, defined as return to baseline or better score in every PostopQRS domain, at 3 days after knee arthroscopy in adult patients.

## Methods

The local institutional ethical review board from the Epworth Hospital Human Research Ethics Committee approved the study (HREC 578–13), which conforms to the ethical guidelines of the 1975 Declaration of Helsinki. Written informed consent was obtained from all patients. The trial was registered prior to participant enrolment at Australian and New Zealand Clinical Trial Registry (ACTRN12613000806763, https://www.anzctr.org.au/Trial/Registration/TrialReview.aspx?ACTRN=12613000806763 Principal investigator Colin Royse, date of registration 22 July 2013). This manuscript adheres to the applicable CONSORT guidelines.[18]

### Design and study patients

In a prospective, single center randomized controlled trial with a two-arm parallel group and a 1:1 allocation ratio, 300 participants were recruited from Epworth HealthCare, Richmond and Box Hill campuses, Melbourne, Australia, during February 2014 until December 2016. Inclusion criteria were age of 18 years or above and undergoing knee arthroscopic surgery under general anesthesia. Participants with additional surgery planned, with allergies to any of the trial drugs, respiratory illness contraindicating volatile anesthesia, or with underlying known cognitive impairment, active psychiatric illness, or insufficient English to complete the survey questions were excluded from the study. Data was collected face-to-face by the research staff whilst participants were in hospital and via the telephone after discharge. There were no protocol changes made during the conduct of the trial.

### Interventions

The intervention was maintenance of anesthesia with either sevoflurane or desflurane, titrated to effect by the treating anesthesiologist.

Standardized care in both groups involved avoidance of any sedative premedication, and induction of anesthesia with propofol and fentanyl. Other aspects of care including use of midazolam, choice of analgesics and airway devices was at the discretion of the anesthesiologist, though the typical practice at these locations includes acetaminophen and non-steroidal anti-inflammatory drugs and airway management via laryngeal mask airway and spontaneous ventilation.

### Outcome

Recovery after surgery was assessed using the “Postoperative Quality of Recovery Scale” (PostopQRS) (S1 Fig) of which details of the construct and validation has been previously published, [16, 19–21] (see www.postopqrs.com). In brief, quality of recovery is measured using a verbal survey tool that measures recovery in five subdomains; physiological, cognitive, nociceptive (pain and nausea), functional (activity-of-daily-living), and emotive (anxiety and depression) recovery. The tool is designed for repeated measurements and can be administered either face-to-face or via the telephone. The learning effect for cognitive tests has been shown to be minimal. [16]

Prior to the operation, the survey is conducted to acquire baseline measurements. Recovery is scored by comparing the postoperative values to baseline values, and performance is dichotomized to “recovered” versus “not recovered” at each of the postoperative time points in each of the five domains if the postoperative score is equal to or greater than the baseline values. Full recovery in every domain implies recovery in all subdomains being assessed at the timepoint. For very early timepoints the ADL domain is not assessed, and for Day 1 and later, the physiological domain is not assessed as participants will have left the hospital. The cognitive subdomain consists of five verbal tests, and the subdomain requires recovery in all five tests. Variance in cognitive performance is a normal event in all people. This was measured in a human volunteer population not undergoing surgery, and a tolerance factor equating to 2 standard deviations of the variability was applied to the cognitive scoring to account for normal variability.[16] This means that patients are allowed to perform a little worse than their baseline performance and still be scored as recovered (for example if a baseline test score for word generation is 10, and the tolerance is 3, then recovery will be scored if the postoperative test score is 7 or greater). However, if the baseline score is less than the tolerance factor, they would automatically be scored as recovered, and therefore, patients with ‘low baseline scores’ cannot be evaluated for cognitive recovery. They are also excluded from Overall Recovery analysis unless they fail to recover in another domain (failure to recover in any domain results in failure to recover in all domains). The incidence of low baseline scores differs amongst populations, being near zero in young volunteers,[16] and 5–15% in orthopedic patients.[21] The tolerance factor is applied as follows: for orientation– 0, Digits forward 2, digits back 1, word recall 3, and word generation task 3.

The PostopQRS was conducted just prior to surgery (baseline), and at 15 minutes, 40 minutes following the last skin stitch by the surgeon, 1 day, 3 days, 1 month, and 3 months after the operation. The physiological domain was measured only at 15 and 40 minutes following the operation. An assessment of patient perspective was performed as a part of the PostopQRS at 1 day, 3 days, 1 month, and 3 months after the operation. This included the patient’s ability to work, ability to undertake daily living activities, clarity of thought, and satisfaction with the anesthetic care received.

The primary outcome was the incidence of Overall Recovery on the PostopQRS survey 3 days after knee arthroscopy, which means that the participant recovers in all items on the PostopQRS survey. ‘Recovery’ requires the participant to have returned to pre-surgery values or better. Secondary outcomes were the incidences of Recovery in the Overall domain and in the 5 subdomains (physiology, nociception, emotion, cognition, and activities of daily living (ADL)) over the 3-month follow-up period. There were no changes to trial outcomes after commencement of recruitment.

### Sample size

The sample-size estimate was based on a pilot data from 20 sevoflurane participants, together with the arthroscopy cohort from a prior observational study using desflurane as the volatile anesthetic (Epworth Hospital HREC LR04310, 19 January 2011).[21] Clinically important trends were observed for Overall Recovery at day 3 (desflurane 35.2% vs sevoflurane 20%). For this study we chose the 3-day time-period so that most participants would no longer require strong analgesics, and inflammation should have largely subsided—both of which could confound small but clinically important differences in recovery at earlier time periods. Using Fisher’s exact method, and based on the difference observed between groups in the pilot study, for Overall Recovery on the PostopQRS at day 3, using a 2-tailed test with 80% power at a 5% significance level, the minimal sample size was 137 for each group, which was increased to 150 patients per group to account for potential non-completions.

### Randomization and blinding

The randomization sequence was generated using web based random sequence generator (https://www.sealedenvelope.com/simple-randomiser/v1/lists) as an unrestricted sequence (without blocking or stratification). A non-participant in the study concealed the allocation using double sealed opaque envelopes. Following recruitment, the envelopes were given to the treating anesthesiologist, who revealed the allocation prior to induction of anesthesia in order to deliver the intervention. The interventions were similar (both volatile anesthetics, but different drugs. Participants, research staff (outcome assessors) and surgeons were blinded to allocation.

### Statistical analyses

The design is a superiority trial with all analyses performed on an intention-to-treat basis. The population analyzed is restricted to adults undergoing knee arthroscopic surgery. Data are presented as means ± standard deviations or as absolute numbers with percentages of patients. The definition of Recovery has been described above and is shown as the proportion of participants recovered at each time point. The probability of Overall Recovery at 3 days (primary outcome) was compared between groups using the Fisher’s exact test. Comparisons of group differences over the whole study period for secondary outcomes were assessed using a Wald’s test on a generalized linear mixed model (GLMM), which was used in order to adjust for within-group variations over time. *P*-values <0.05 were considered statistically significant, all *p*-values are two-sided. Descriptive data were stored in Microsoft Excel 2016 (Microsoft Corp., CA, USA) and for statistical analyses we used Stata/IC 12.1 for Mac (Stata Corp., TX, USA). No interim analyses were performed, and no stopping rules defined. No ancillary analyses were performed due to the limited sample size.

## Results

**Fig 1:**
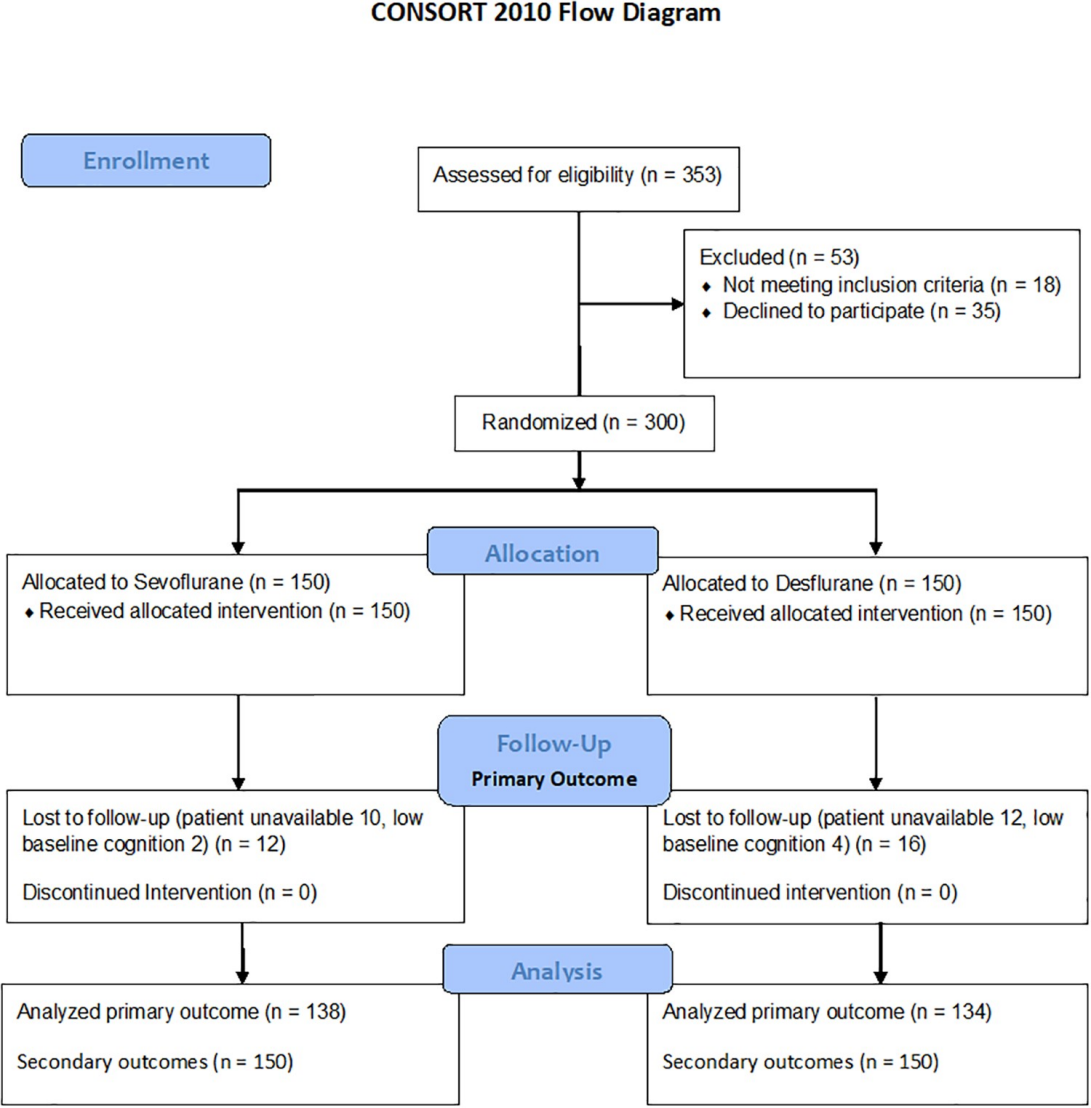
CONSORT participant flowchart. Participant flowchart.

In the period from February 2014 to December 2016, 300 patients were enrolled as displayed in detail in Fig 1. The trial stopped after all patients were enrolled. The enrolled patients had complete baseline assessments of which 6 patients had low baseline cognitive scores. No harms or unintended events occurred for any participants.

Patient demographics are shown in Table 1, whereas operative details and drugs administered are displayed in Table 2. There was a difference in the age between the two groups, with an older cohort in the participants who had received desflurane, 51.7 ± 14.1 compared to sevoflurane, 47.3 ± 13.5, p = 0.006. There was also a greater body mass index seen in the desflurane group, 29.4 ± 6.2 compared to the sevoflurane group, 27.7 ± 4.6, p = 0.008. There was a tendency towards more ASA 1 patients in the sevoflurane group and correspondingly more ASA 2 patients in the desflurane group. The mean duration of anesthesia, from induction to cessation of the volatile anesthetic was 24.9 ± 11.1 minutes for sevoflurane and 23.3 ± 8.3 minutes for desflurane, p = 0.156, and the mean time to eye-opening from the last skin stitch to the patient opening their eyes was 14.3 ± 7.7 minutes for sevoflurane and 13.9 ± 10.1 minutes for desflurane, p = 0.699.

**Table 1:** Characteristics of patients undergoing knee arthroscopic surgery.

	*Sevoflurane* *n = 150*	*Desflurane* *n = 150*
**Demographics**		
Age (years)	47.3 ± 13.5	51.7 ± 14.1
Male, n (%)	95 (63.3)	99 (66)
Body mass index, kg/m^2^	27.7 ± 4.6	29.4 ± 6.2
**ASA**^a^ **status**		
ASA I	97 (64.7)	74 (49.3)
ASA II	48 (32)	66 (44)
ASA III	5 (3.3)	10 (6.7)
**Years in full-time education (years)**	14.8 ± 2.9	14.6 ± 3.7
**Alcohol units per day (standard drinks)**	5.3 ± 6.4	4.9 ± 6
**Smoking status**		
Active smokers, n (%)	13 (8.7)	12 (8)
Former smokers, n (%)	47 (31.3)	53 (35.3)
Non-smokers, n (%)	90 60)	85 (56.7)

Data reported as means ± standard deviations or absolute numbers and percentages of patients.

^a^ASA, American Society of Anesthesiology Physical Classification System

**Table 2:** Operative details and drugs administered to patients undergoing knee arthroscopic surgery.

	*Sevoflurane* *n = 150*	*Desflurane* *n = 150*
**Airway device**		
None, n (%)	4 (2.7)	2 (1.3)
Laryngeal mask airway, n (%)	146 (97.3)	145 (96.7)
Endotracheal tube, n (%)	0 (0)	2 (1.3)
**Ventilation method**		
Spontaneous breathing, n (%)	103 (68.7)	91 (60.7)
Pressure support ventilation, n (%)	20 (13.3)	22 (14.7)
Mechanical ventilation, n (%)	27 (18)	36 (24)
**Pre-medication**		
None, n (%)	142 (94.7)	141 (94)
Acetaminophen, n (%)	5 (3.3)	4 (2.7)
Midazolam, n (%)	2 (1.3)	5 (3.3)
Opiate, n (%)	1 (0.7)	0 (0)
**Anesthetic induction**		
Propofol, n (%)	150 (100)	150 (100)
**Intraoperative benzodiazepines**		
None, n (%)	64 (42.7)	75 (50)
Midazolam, n (%)	86 (57.3)	74 (49.3)
**Cardiovascular drugs**		
None, n (%)	121 (81)	122 (82)
Inotropes, n (%)	1 (1)	1 (1)
Vasopressors, n (%)	9 (6)	9 (6)
Vasodilators, n (%)	3 (2)	1 (1)
Atropine, n (%)	7 (5)	11 (7)
Ephedrine, n (%)	1 (1)	1 (1)
ß-blocker, n (%)	2 (1)	7 (5)
Metaraminol, n (%)	0 (0)	1 (1)
Glycopyrrolate, n (%)	9 (6)	4 (3)
**Intraoperative analgesics**		
None, n (%)	0 (0)	1 (1)
Acetaminophen, n (%)	8 (5)	11 (7)
NSAID^b^, n (%)	109 (73)	117 (79)
Tramadol, n (%)	26 (17)	34 (23)
Fentanyl, n (%)	130 (87)	118 (79)
Morphine, n (%)	28 (19)	32 (21)
Hydromorphone, n (%)	0 (0)	1 (1)
Alfentanil, n (%)	1 (1)	2 (1)
Oxycodone, n (%)	5 (3)	9 (6)
LA^b^ infiltration, n (%)	88 (59)	82 (55)
**Postoperative analgesics**		
None, n (%)	4 (3)	6 (4)
Acetaminophen, n (%)	23 (15)	23 (15)
NSAID^a^, n (%)	25 (17)	23 (15)
Tramadol, n (%)	4 (3)	1 (1)
Codeine, n (%)	6 (4)	4 (3)
Fentanyl, n (%)	96 (64)	85 (57)
Morphine, n (%)	39 (26)	43 (29)
Pethidine, n (%)	0 (0)	1 (1)
Oxycodone, n (%)	9 (6)	12 (8)
LA^b^ infiltration, n (%)	11 (7)	7 (5)
**Antiemetics**		
None, n (%)	11 (7)	11 (7)
Dexamethasone, n (%)	116 (77)	96 (64)
Ondansetron, n (%)	45 (30)	47 (32)
Granisetron, n (%)	36 (24)	30 (20)
Metoclopramide, n (%)	20 (13)	23 (15)
Droperidol, n (%)	4 (3)	1 (1)
Antihistamine, n (%)	1 (1)	1 (1)
**Other drugs administered**		
N_2_O, n (%)	9 (6)	13 (9)
Muscle relaxant, n (%)	0 (0)	1 (0)
Reversal agent, n (%)	0 (0)	1 (0)
Blood products, n (%)	0 (0)	1 (0)

Data reported as means ± standard deviations or absolute numbers and percentages of patients.

^a^NSAID, non-steroidal anti-inflammatory drug

^b^LA, local anesthetic

**Fig 2:**
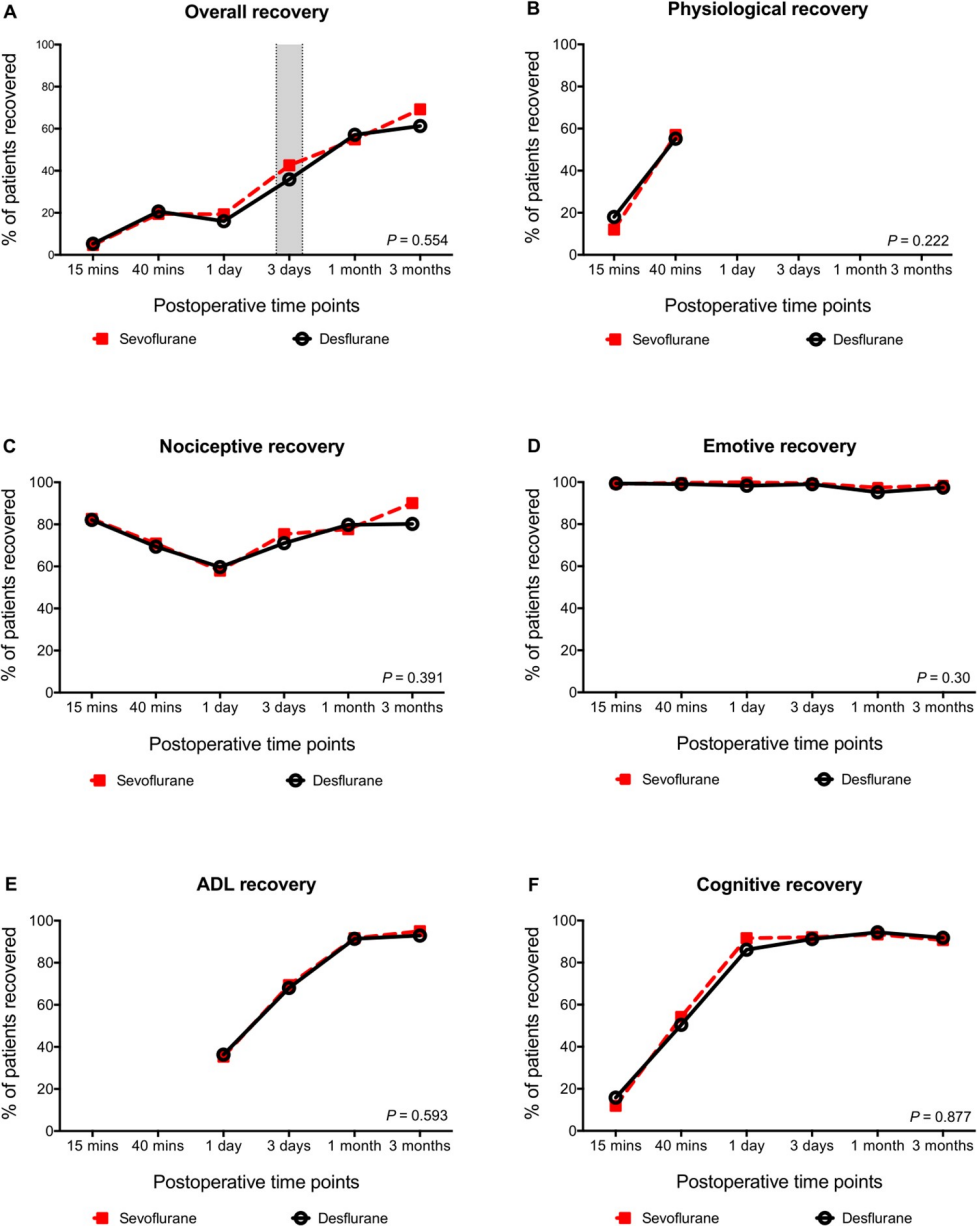
Recovery profiles following knee arthroscopy under either sevoflurane or desflurane general anesthesia. Recovery profiles following knee arthroscopy under either sevoflurane or desflurane general anesthesia. The graphs display the percentages of patients recovered in the two groups at each timepoint. (**A**) shows the Overall Recovery profile (primary endpoint marked with grey), whereas (**B** to **F)** show the five subdomains. ADL, activity of daily living.

Quality of Recovery over time is illustrated in Fig 2. As seen, Recovery improved over the follow-up period in both groups in the Overall domain as well as all subdomains. Overall Recovery improved over the follow-up period in both groups but was not different at day 3 (sevoflurane 43% vs. desflurane 37%, *p* = 0.314 absolute difference 6%). There was no difference between the groups in Overall Recovery over the 3-month period, *p* = 0.554. Likewise, we found no differences in Recovery over time in the five subdomains: physiological Recovery, *p* = 0.222; nociceptive Recovery, *p* = 0.391; emotive Recovery, *p* = 0.300; ADL Recovery, *p* = 0.593; and cognitive Recovery, *p* = 0.877.

**Fig 3:**
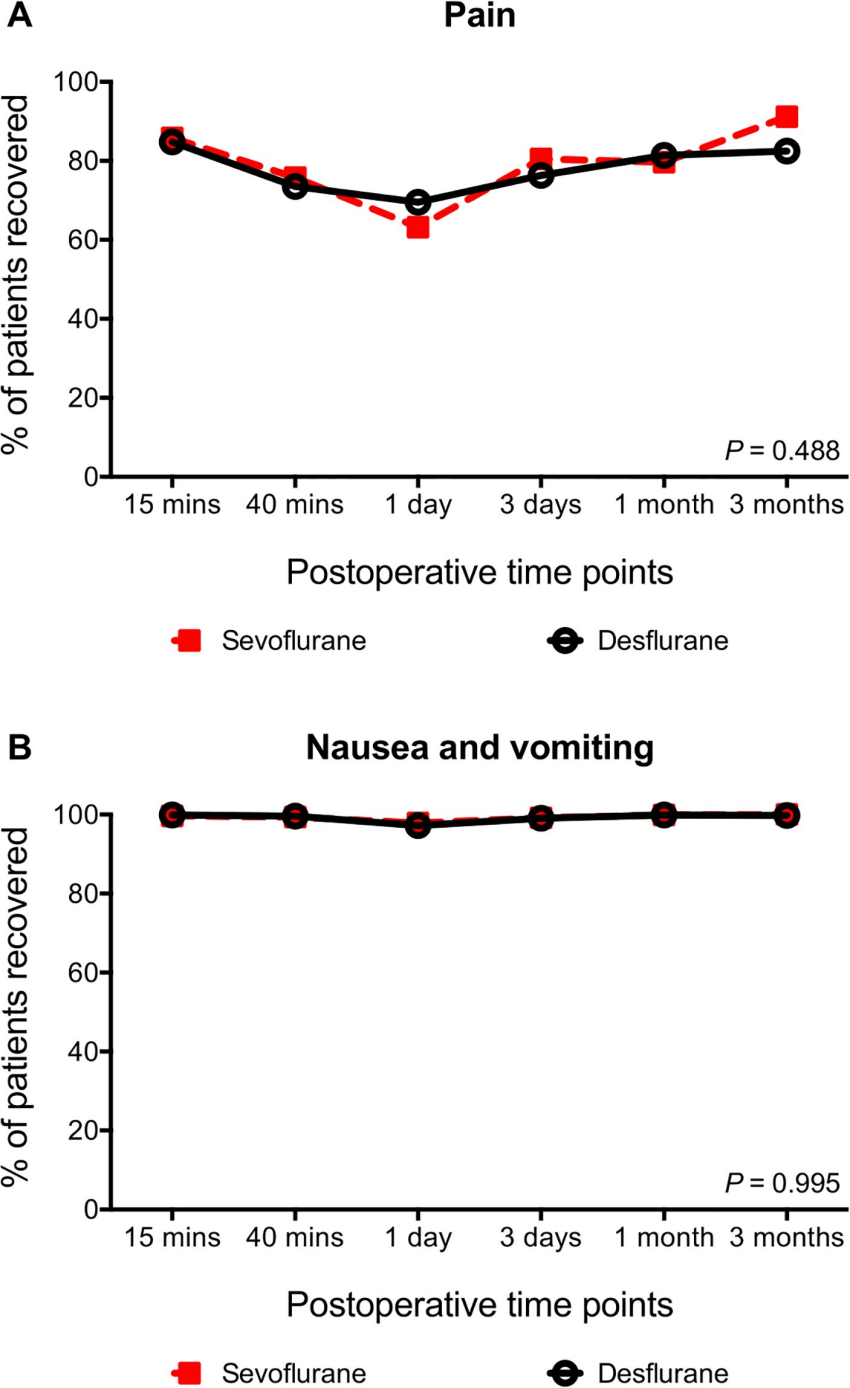
Recovery profiles following knee arthroscopy under either sevoflurane or desflurane general anesthesia. Recovery profiles following knee arthroscopy under either sevoflurane or desflurane general anesthesia in each of the items comprising the nociceptive subdomain. (**A)** shows the percentages of patients recovered in terms of pain, whereas (**B)** shows the percentages of patients recovered in terms of nausea and vomiting.

A breakdown on the items comprising the nociceptive subdomain is displayed in Fig 3, and as displayed, there were no differences in either pain, *p* = 0.488, nor nausea and vomiting, *p* = 0.995, between the two groups.

**Fig 4:**
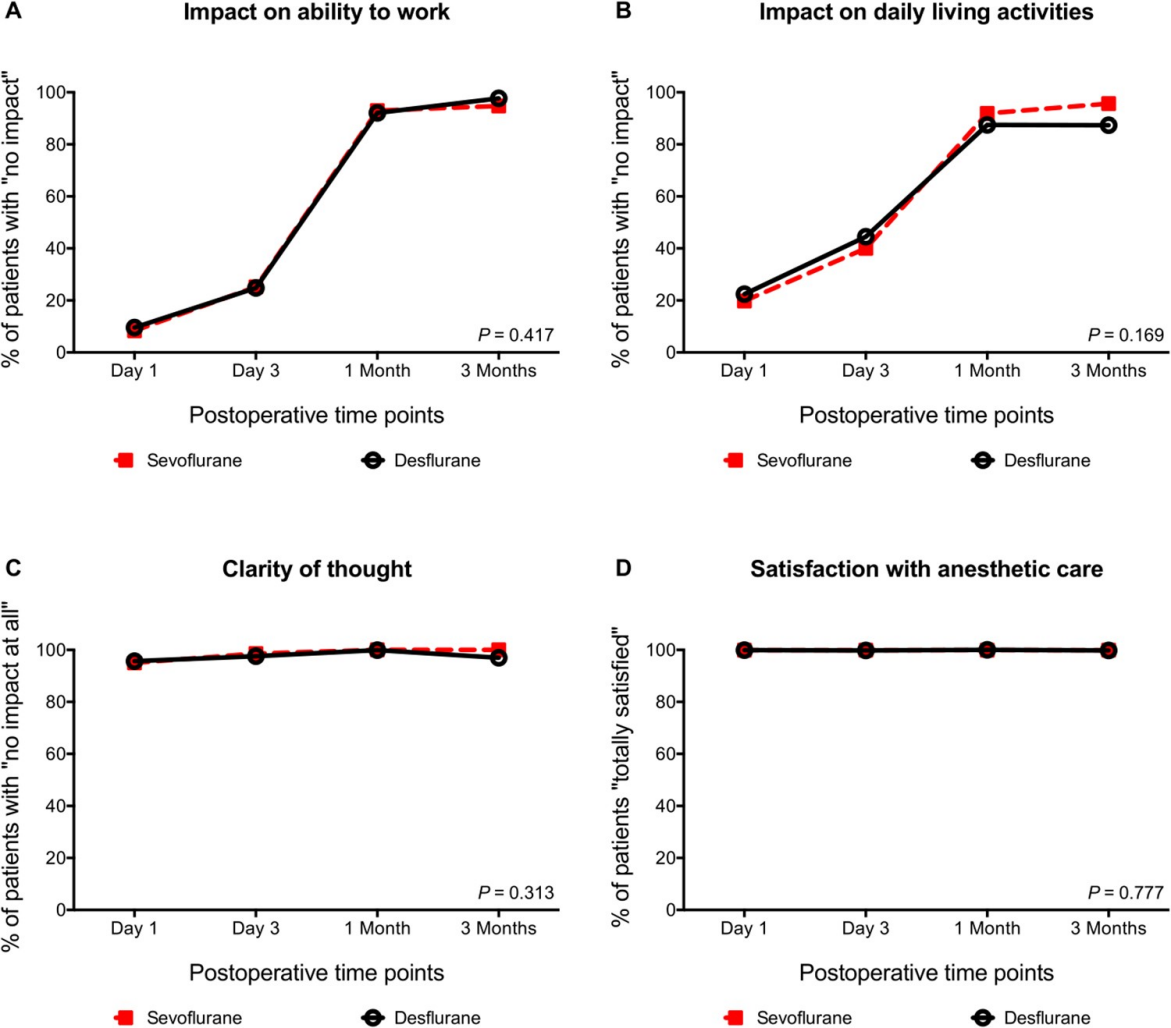
Patient perspective following knee arthroscopy under either sevoflurane or desflurane general anesthesia. Patient perspective following knee arthroscopy under either sevoflurane or desflurane general anesthesia. **(A)** shows the percentages of patients reporting “mild or no impact” on ability to work, (**B)** shows the percentages of patients reporting “mild or no impact” on ability to undertake daily living activities, (**C)** shows the percentages of patients reporting “no impact at all” on clarity of thought, and **(D)** shows the percentages of patients reporting “satisfied or totally satisfied” with anesthetic care.

Patient perspective in terms of ability to work, ability to undertake daily living activities, clarity of thought, and satisfaction with anesthetic care is displayed in Fig 4. As seen, there were no differences in patient perspective between the groups in any of the four items. All patients were satisfied with the anesthetic care.

## Discussion

This prospective, randomized study showed that there are no differences for quality of recovery between sevoflurane and desflurane general anesthesia for knee arthroscopy measured using the PostopQRS in adult participants in either Overall Recovery or individual Recovery domains. The incidence of nausea and vomiting was very low in both groups, which is contrary to common view among anesthesiologists not to use volatiles in patients with a history of nausea. As these findings cannot be directly translated to longer surgery or other populations, further comparative studies are needed to assess quality of recovery across multiple domains with long-term follow-up after longer duration and different surgical procedures.

Previous studies on postoperative recovery after short peripheral surgery have assessed patients only in the immediate recovery period from arrival at the post-anesthesia care unit to time of discharge (i.e. no later than 24 hours after the procedure).[5, 6, 8, 22] Nevertheless, the available data are generally consistent with the findings in this study showing high rates of physiological recovery shortly after the procedure. In the current study, however, we used the PostopQRS, which is a tool designed for repeated measurements and which can be administered via the telephone allowing recovery assessment over a longer duration.[16]

In this study, we assessed quality of recovery across multiple domains and as demonstrated, the key contributor to failure to recover at day 3 was postoperative pain and subsequently, inability to perform daily living activities. However, there was no difference in the incidence of pain between the two volatiles, which is in accordance with previous studies assessing pain prior to discharge. [6, 7, 23] Similarly, we found no differences in cognitive Recovery between sevoflurane and desflurane, which is also consistent with studies showing no differences in the immediate postoperative period. [7, 8, 23–25] Nevertheless, the current study went further to describe potential differences in intermediate and long-term recovery. Day 3 was chosen for the primary outcome, as the patients would be mobile and likely to have ceased strong analgesics (which may confound subtle differences in recovery—especially in the cognitive domain). We identified that even at 3 months after surgery, there was a substantial proportion of participants who had not Recovered, with pain (worse than before surgery) being the most important contributor. We are unable to determine why this occurs, but it is possible that the pathology leading to the arthroscopic procedure was not corrected by the procedure and that some of these patients may proceed to knee arthroplasty. The benefit or futility of arthroscopy for specific cohorts is an important question which we are unable to answer with this study.

Our data are generally in accordance with the available comparative data between sevoflurane and desflurane on postoperative nausea and vomiting,[5, 22, 24] though the incidence was low in both groups. This may be due to routine use of dual prophylactic antiemetic therapy, which could overcome the propensity of volatile anesthetics to induce nausea. This is a noteworthy observation as it is common among anesthesiologists to avoid volatiles in patients with a history of nausea, even for short surgical procedures.

### Limitations

The sample size was based on the difference observed in our non-randomized pilot study. The incidence of recovery observed in this trial for sevoflurane was substantially higher than that observed in the pilot study, which affects the sample size predictions. The sample size would allow a detection of an absolute difference of 15% in primary outcome. A larger sample size could have potentially detected a smaller but maybe clinically important difference between agents. However, results showed no potential trends and no clinically important differences were revealed ins secondary outcomes. The sample size would allow a detection of an absolute difference of around 15%, but too small to detect a smaller but potentially clinically important difference between agents. However, our data showed no trends of group separation at all and no clinically important differences were revealed.

We tested a specific cohort of patients—adults undergoing brief peripheral joint surgery. The use of an unrestricted randomization sequence could introduce allocation bias, though we believe this was mitigated by the use of a single operative procedure, one hospital only and a small cohort or anesthesiologists and surgeons. Our findings cannot be extrapolated to longer surgery or other populations without further research, which include comparative studies assessing quality of recovery across multiple domains with long-term follow-up. Other than the intervention, we did not control the other components of anesthesia or analgesia. The use of other drugs such as midazolam, however, was similar between groups and potential confounding mitigated by randomization. There is potential for performance bias due to the number of anesthesiologists and surgeons participating in the trial, but this is mitigated by use of a single center design where the surgical and anesthetic practice is similar between clinicians. Postoperative analgesia was determined by the treating anesthesiologist rather than according to a prespecified protocol. However, the distribution of analgesics was not different between groups. There were multiple research staff involved in the collection of the data leading to the possibility of reporting bias, however all research staff were trained in conducting the PostopQRS in a standardized format. Furthermore, detection bias was mitigated through blinding of all research clinicians involved except for the treating anesthesiologist. There were few refusals or exclusions and attrition bias was low (around 10%).

## Conclusion

No significant difference in the quality of recovery scale could be shown using sevoflurane or desflurane general anesthesia after knee arthroscopy in adult participants.

## Supporting information

S1 Checklist CONSORT checklist. (DOC)

S1 Protocol RCT578-13 protocol ‘a randomized trial of desflurane or sevoflurane on postoperative quality of recovery after knee arthroscopy’. (DOCX)

S1 Fig Postoperative Quality of Recovery Scale. Postoperative Quality of Recovery Scale.
(PDF)
