# Supplementary material for: A randomized trial of desflurane or sevoflurane on postoperative quality of recovery after knee arthroscopy
Source: PLoS One. 2019 Aug 5;14(8):e0220733. doi: 10.1371/journal.pone.0220733 (PMC6681958; doi:10.1371/journal.pone.0220733)
Supplement: S1 Protocol — (DOCX) [file pone.0220733.s002.docx]

## Clinical Research Proposal

###### Date of draft: Version 2 dated 18 July 2013

**Study protocol**

A randomized trial of sevoflurane versus desflurane on the quality of recovery following knee arthroscopy

### **Principal investigators**: Professor Colin Royse

Study sites: The study will be conducted at the Epworth Healthcare, Richmond and Box Hill campuses, Melbourne, Victoria, Australia.

### **Background**

Recovery following general anaesthesia is a complex issue confounded by the type of surgery, inflammation, different anaesthetic drugs and techniques, patient co-morbidities, and differing patient and clinician perceptions of what constitutes good recovery.

Recovery is not a single entity but rather covers many aspects or domains such as physiological recovery, pain and nausea, emotion and mood, return to normal life or work activities, and cognitive function. It is an entity that is difficult to quantify, which then makes it difficult to study in a systematic manner. For anaesthetists, poor recovery is often relayed by the surgeon days or weeks after the event, and it is usually categorized as an adverse outcome.

Current research tools such as the Aldrete ([1](#_ENREF_1)) or the QoR ([2](#_ENREF_2), [3](#_ENREF_3)) scales, focus on early physiological recovery, or the immediate perioperative period. These recovery scores are not sensitive enough to measure the rate of recovery (change over time), and have not been designed for repeated measures. They are also inadequate to identify poor cognitive recovery.

In 2007, an international group of anaesthetists and neuropsychologists formed an advisory board to create a new quality of recovery scale. The aim was to produce a tool that was simple to perform, but sensitive enough to detect change in multiple domains of recovery over time. The validation experiments have included over 700 patients, and this work has been accepted and is published in Anesthesiology ([4](#_ENREF_4)). It is called the Postoperative Quality Recovery Scale (PQRS). Six domains of recovery are identified: physiological, nociceptive (pain and nausea) emotive (anxiety and depression), functional recovery (return of activities of daily living), self assessed recovery, and cognitive recovery. The scale is completed prior to surgery to provide baseline values, and then repeated at 15 minutes, 40 minutes, 1 and 3 days, and 3 months after the completion of anaesthesia (typically after the last surgical stimulation). Recovery is broadly defined as return to baseline or better.

.

One of the most important benefits of the PQRS scale is that it enables recovery to be quantified and measured. This makes it possible to compare different interventions with the express purpose of developing specific interventions to improve quality of recovery. The PQRS offers a tool to provide the recovery process to be examined. There are no other tools in existence that provide a comprehensive, sensitive assessment of the multiple aspects or domains of recovery, and is yet relatively simple to perform.

Much research has been conducted on the general recovery from knee surgery in relation to mobility and quality of life ([5](#_ENREF_5), [6](#_ENREF_6)) as well as pain relief ([7](#_ENREF_7), [8](#_ENREF_8)) and risk of complications such as deep venous thrombosis ([9](#_ENREF_9), [10](#_ENREF_10)). In addition there has been a significant amount of research that has considered the impact of both hip and knee replacement surgery on cognition where the mechanism of cognitive dysfunction is considered to be lipid microemeboli that have been detected by Trancranial Doppler (TCD) ([11](#_ENREF_11), [12](#_ENREF_12)).

In routine care patients are frequently discharged home on the same day or following day with arthroscopy, which means that they are not assessed by medical or nursing staff until they return for follow-up review, or represent with complications. It is unclear whether long-term recovery (especially cognitive recovery) can be affected by brief surgery such as arthroscopy. The validation data for the PQRS is suggestive that cognitive quality of recovery may be impaired even with brief anaesthesia and surgery.

General anaesthesia induces a reversible but profound state of coma. Recovery from that can vary considerable between individuals as described above. Less is known about the potential contribution from the anaesthetic drugs to the process of complete or incomplete recovery (especially cognitive recovery), largely due to the paucity of randomized trials.

Sevoflurane and desflurane are the two most commonly used anaesthetics in Australia from the “inhalational anaesthetic“ class of drugs. Although they differ in potency, they both produce equivalent depths of anaesthesia. From a pharmacokinetic perspective, desflurane works more quickly than sevoflurane and is eliminated more rapidly as well, leading to more rapid emergence from anaesthesia. Desflurane also undergoes minimal biotransformation (0.02%), whereas sevoflurane undergoes greater biotransformation (5%). In human volunteers undergoing prolonged anaesthesia with either sevoflurane or desflurane, the desflurane group recovered in multiple domains much more rapidly than sevoflurane ([13](#_ENREF_13)). Furthermore, it has been shown that early recovery parameters such as return of pharyngeal reflexes and wakefulness are faster with desflurane ([14](#_ENREF_14), [15](#_ENREF_15)). What is apparent, however, is that there is very little variability in these parameters with desflurane, but considerable variability with sevoflurane leading to a much wider range of recovery times. In the paediatric population, the incidence of emergence agitation is higher with sevoflurane than desflurane([16](#_ENREF_16)). Desflurane, however is more irritant (can lead to coughing) at high concentration, and heart rate can increase if a high concentration is rapidly introduced ([17](#_ENREF_17)). At the typical anaesthetic concentrations used in surgery, however the drugs are comparable in terms of airway irritability and haemodynamic responses ([17](#_ENREF_17), [18](#_ENREF_18)).

It is unknown, however, if there are significant differences in the rate of recovery across the multiple domains that this involves, over a longer time period between these two inhalational anaesthetics, in an adult population when administered for a relatively brief duration.

### **Clinical significance**

Quality of recovery is an emerging field within anaesthesia of great importance. Although large outcome studies are very important in anaesthesia, there is a changing focus from “mortality and morbidity studies”, to quality of recovery. The reason is that the frequency of mortality is now very low with the result that few interventions will further reduce mortality and in any event very large numbers will be required to demonstrate any improvements in surgery and anaesthesia with mortality as an outcome. However early data on the PQRS as well as clinical reports indicate that the quality of recovery is often poor in many patients, and yet these are not identified by the treating anaesthetist. There are implications for the individual patient, for the practice of anaesthesia, and for the community (such as safe return to work or to driving).

Knee arthroscopy is often performed as an outpatient (day) procedure, and therefore may be perceived as “minor surgery”. However, for some patients the quality of recovery including cognitive decline may be more profound than would be expected from brief peripheral surgery. The role of the anaesthetic agent may have an important influence on this recovery. It is frequently assumed by anaesthetists that anaesthetics from the same class should produce similar effects when administered for a brief anaesthetic, but there are insufficient data to validate that assumption. This study will help anaesthetists to choose the anaesthetic that is best for their patients undergoing brief surgery.

Pilot data

At the Epworth Healthcare campuses, we have conducted an observational study investigating the effects of age (young <40 yrs and older >65 years) on recovery in patients having arthroscopy surgery and comparing the older group to knee replacement surgery (HREC # 46309). To reduce the risk of confounding bias, all patients received the same anaesthetic (desflurane).

However, we conducted a pilot study arm with sevoflurane as the general anaesthetic agent. We recruited 10 patients in each of the young and older cohorts. Other aspects of the study conduct were identical to the patients receiving desflurane anaesthesia. In brief, Patients were induced with propofol, and maintained on wither desflurane or sevoflurane according to allocation. Intraoperative and postoperative opiates were used for analgesia. Nitrous oxide anaesthesia was not used. Patients were typically discharge on the same day of surgery. PQRS measurements were performed at Baseline n the day of surgery, 15 and 40 minutes, 1 and 3 days, and 3 months after the end of anaesthesia.

Pilot study results:

Due to the small numbers in each of the young and old groups in the sevoflurane arms, all patients were combined to compare the recovery profiles of desflurane and sevoflurane. However, the proportion of patients in young and old ages were the same for both anaesthetics. 122 patients are included in the desflurane group and 20 in the sevoflurane group.

The figure below shows recovery between groups for all domains, as well as the individual recovery domains.

The P value displayed is derived using the Cochran Mantel Haenzel test for repeated

measurement of proportions, and tests the global hypothesis of whether a difference exists in the recovery profiles over time between the two agents. P<0.05 was used to define significance.

Caution must be exercised in interpreting the P value, due to the small n in the sevofluane group compared to the desflurane, and there is high chance of Type II error where there is a P>0.05. Rather, place emphasis on the presence of absence of differences in the recovery profiles, as an indicator of effect size.

*A. Physiological recovery domain for Time 15 and 40 minutes. B. Cognitive recovery profile using the revised cognition scoring method. C. Nociceptive domain includes pain and nausea. D. ADL is activities of daily living and tests the ability to eat, walk, stand and dress. E. Emotive recovery tests feelings of anxiety and depression. F. Overall recovery is present when there is recovery on all of the domains (A-E).*

Of note, there is improved nociceptive recovery with desflurane (P<0.05). All other domains were not statistically different, but there were reasonable effect size differences for Cognitive recovery on day 1 (desflurane 78% vs sevoflurane 56%), and for all domains recovery on day 3 (desflurane 35.2% vs sevoflurane 20%).

### **Objectives:**

1. To identify whether the rate/quality of recovery is affected by sevoflurane versus desflurane when used as the primary anaesthetic for knee arthroscopy surgery.

### **Hypothesis**

1. The null hypothesis is that there is no difference in recovery measured using the PQRS, in patients undergoing knee arthroscopy under general anaesthesia with either sevoflurane or desflurane as the primary anaesthetic.

### **Study design**

Parallel randomized trial with allocation ration 1:1.

### **Study environment**

The study will be conducted in a multi site private hospital, which has an active orthopaedic unit (Epworth HealthCare Richmond or Box Hill, Victoria, Australia). It is anticipated that a 24 month study period will be required. The institution conducts approximately 1500 orthopaedic operations per year, of which knee surgery constitutes approximately 25% of operations. The patient cohort is selected so as not to compete for patients with a current study, as well as to control for age.

### **Eligibility criteria**

Inclusion:

1. Adult patients undergoing knee arthroscopy surgery under general anaesthesia

Exclusion:

1. Patients, who are not fluent in English, will be excluded, as they may be unable to answer the recovery questionnaire adequately.
2. Patients undergoing regional anaesthesia only

### **Interventions**

**Common management for both groups wil include the following:**

1. Pre-medication other than oral analgesic (such as paracetamol) will not be used.

2. Induction will be with intravenous propofol, including co-induction consisting of fentanyl 25-100 micrograms and midazolam 1-5 mg. Antimemetics including dexamethasone and 5HT_3_ antagnists (such as ondensetron) may be administered by the treating anaesthetist.

3. Analgesia will consist of intraoperative opiates, 1 g paracetamol qid, intravenous opiates during early recovery (such as 1.v. fentanyl), Morphine 2.5-10mg sc 3 hourly, or oxynorm 10-20mg 6 hourly will be used for breakthrough analgesia. NSAID’s including Cox II inhibitors (such as paracoxib 40mg i.v. amy be used.

4. Patients will not be included in the study if the treating anaesthetist considers that a regional anaesthetic rather than a general anaesthetic would be in the best interest of the patient. This would most likely be a spinal anaesthetic.

**Intervention drug.**

Following induction of general anaesthesia either desflurane or sevoflurane will be introduced as the maintenance anaesthetic and titrated to maintain an adequate clinical depth of anaesthesia for the remainder of the operation. Typically this will involve anaesthetic concentrations of 6% desflurane and 2.4% sevoflurane, reflecting the different potencies of the drugs. If Bispectral index monitoring (depth of anaesthesia monitor) is available, the target range is 40-60.

### **Outcome measurements for both studies**

**Primary outcome**:

Quality of recovery, as measured by the postoperative quality recovery scale (PQRS) at Day 3 for recovery in all of the individual recovery domains (all domains recovery).

**Secondary outcome**:

1. Recovery for all domains and within domains at the other time points of measurement (15 minutes, 40 minutes 1 day, 3 days and 3 months following cessation of anaesthesia). The domains of recovery are physiological, nociceptive, emotive activities of daily living, cognitive and overall patient perspective.

**Study assessments protocol**

Patients will be recruited prior to but on the same day as surgery. Baseline measurements using the PQRS will be performed prior to premedication.

Measurements will then be performed using the PQRS battery at 15 minutes, 40 minutes, one day, three days and three months following surgery. This is the same testing paradigm as we have used in the validation study of the PQRS. Whilst in hospital, testing will be conducted face-to-face, whereas following discharge from hospital; these will be conducted via telephone interview.

### **Study size**

Sample-size estimates are based on the pilot data for all domains recovery at day 3 (desflurane 35.2% vs sevoflurane 20%) and using Fisher’s exact method to determine a difference of 10% difference in the total score using a 2-tailed test with 80% power at a *P*=0.05 significance. The minimal sample size is 137 for each group. This will be increased to 150 patients per group to account for potential non-completions.

### **Statistical methods**

Continuous variables will be analysed by Student’s t test for independent samples, or repeated measures ANOVA for multiple measurements over time. The primary endpoint will be assessed using Fisher’s exact test. Recovery profiles over time will be tested using the Cochran Mantel Haenzel test for repeated measurement of proportions, and will test the global hypothesis for difference between groups over time.

### **Ethical issues**

This study will be reviewed by the Epworth HealthCare HREC. All patients will receive a written plain language statement and will provide informed written consent.

Both desflurane and sevoflurane are commonly used general anaesthetics in Australia. If the participating anaesthetist believes that the anaesthetic regimen is inappropriate, or that the patient requests regional anaesthesia, then the patient will not be recruited into the study. Each PQRS measurement takes approximately 5-10 minutes, and is not onerous or stressful to the patient. However, if the patient does not wish to participate further, then they may withdraw from the study. Measurements performed after discharge from hospital are conducted via telephone interviews and do not require the patient to return to the hospital or other location. The interviews are arranged between the researcher and patient at a time of mutual convenience.

### **Randomization**

The randomization sequence will by produced using a computer generated randomization sequence. Concealment will be by placing the card containing the allocation information in double opaque sealed envelopes, and concealment will be maintained until after recruitment and the patients is admitted into the operating theatre. The treating anaesthetist will then open the envelopes to reveal the allocation. A non-participant in any process of the study will perform preparation of the envelopes. A copy of the randomization sequence will be stored in a separate databank, which is password protected and not available to the investigators until the study is complete.

### **Blinding**

Due to the nature of the drugs, it is not possible to blind the treating anaesthetist to the allocation, but the patient and study investigators who collect the PQRS data will be blinded to allocation.

### **Anticipated time-line**

The aim is to recruit 3 patients per week. That timeline for the study schedule is listed below.

| **Objective** | **October 2012 to March 2013** | **March – May 2013** | **June 2013 to**  **August 2014** | **Aug 2014 to Dec 2014** | **Dec 2014 to March 2015** |
| --- | --- | --- | --- | --- | --- |
| Ethics | XXXXXXXX |  |  |  |  |
| Recruit 30% |  | XXXXXXXXX |  |  |  |
| Recruit 100% |  |  | XXXXXXXXX |  |  |
| Complete 3 month follow up |  |  |  | xxxxxxx |  |
| Analysis and manuscript |  |  |  |  | XXXX |

### References

1. Aldrete JA, Kroulik D. A postanesthetic recovery score. Anesth Analg. 1970;49(6):924-34. Epub 1970/11/01.

2. Myles PS, Reeves MD, Anderson H, Weeks AM. Measurement of quality of recovery in 5672 patients after anaesthesia and surgery. Anaesth Intensive Care. 2000;28(3):276-80. Epub 2000/06/15.

3. Myles PS, Weitkamp B, Jones K, Melick J, Hensen S. Validity and reliability of a postoperative quality of recovery score: the QoR-40. Br J Anaesth. 2000;84(1):11-5. Epub 2000/03/31.

4. Royse CF, Newman S, Chung F, Stygall J, McKay RE, Boldt J, et al. Development and feasibility of a scale to assess postoperative recovery: the post-operative quality recovery scale. Anesthesiology.113(4):892-905. Epub 2010/07/06.

5. Johnston L, MacLennan G, McCormack K, Ramsay C, Walker A. The Knee Arthroplasty Trial (KAT) design features, baseline characteristics, and two-year functional outcomes after alternative approaches to knee replacement. J Bone Joint Surg Am. 2009;91(1):134-41. Epub 2009/01/06.

6. Razmjou H, Schwartz CE, Yee A, Finkelstein JA. Traditional assessment of health outcome following total knee arthroplasty was confounded by response shift phenomenon. J Clin Epidemiol. 2009;62(1):91-6. Epub 2008/12/20.

7. Fagan DJ, Martin W, Smith A. A randomized, double-blind trial of pre-emptive local anesthesia in day-case knee arthroscopy. Arthroscopy. 2003;19(1):50-3. Epub 2003/01/11.

8. Vranken JH, Vissers KC, de Jongh R, Heylen R. Intraarticular sufentanil administration facilitates recovery after day-case knee arthroscopy. Anesth Analg. 2001;92(3):625-8. Epub 2001/02/28.

9. Alexander G, Gustafson A, Wise G. Comparison of warfarin use in total knee replacement vs total hip replacement. Orthopedics. 1997;20(2):117-20. Epub 1997/02/01.

10. Brookenthal KR, Freedman KB, Lotke PA, Fitzgerald RH, Lonner JH. A meta-analysis of thromboembolic prophylaxis in total knee arthroplasty. J Arthroplasty. 2001;16(3):293-300. Epub 2001/04/18.

11. Rodriguez RA, Tellier A, Grabowski J, Fazekas A, Turek M, Miller D, et al. Cognitive dysfunction after total knee arthroplasty: effects of intraoperative cerebral embolization and postoperative complications. J Arthroplasty. 2005;20(6):763-71. Epub 2005/09/06.

12. Koch S, Forteza A, Lavernia C, Romano JG, Campo-Bustillo I, Campo N, et al. Cerebral fat microembolism and cognitive decline after hip and knee replacement. Stroke. 2007;38(3):1079-81. Epub 2007/01/27.

13. Eger EI, 2nd, Bowland T, Ionescu P, Laster MJ, Fang Z, Gong D, et al. Recovery and kinetic characteristics of desflurane and sevoflurane in volunteers after 8-h exposure, including kinetics of degradation products. Anesthesiology. 1997;87(3):517-26. Epub 1997/10/08 22:28.

14. McKay RE, Malhotra A, Cakmakkaya OS, Hall KT, McKay WR, Apfel CC. Effect of increased body mass index and anaesthetic duration on recovery of protective airway reflexes after sevoflurane vs desflurane. Br J Anaesth.104(2):175-82. Epub 2009/12/29.

15. McKay RE, Large MJ, Balea MC, McKay WR. Airway reflexes return more rapidly after desflurane anesthesia than after sevoflurane anesthesia. Anesth Analg. 2005;100(3):697-700, table of contents. Epub 2005/02/25.

16. Mayer J, Boldt J, Rohm KD, Scheuermann K, Suttner SW. Desflurane anesthesia after sevoflurane inhaled induction reduces severity of emergence agitation in children undergoing minor ear-nose-throat surgery compared with sevoflurane induction and maintenance. Anesth Analg. 2006;102(2):400-4. Epub 2006/01/24.

17. Arain SR, Shankar H, Ebert TJ. Desflurane enhances reactivity during the use of the laryngeal mask airway. Anesthesiology. 2005;103(3):495-9. Epub 2005/09/01.

18. Eshima RW, Maurer A, King T, Lin BK, Heavner JE, Bogetz MS, et al. A comparison of airway responses during desflurane and sevoflurane administration via a laryngeal mask airway for maintenance of anesthesia. Anesth Analg. 2003;96(3):701-5, table of contents. Epub 2003/02/25.
