# Supplementary material for: A randomized trial of desflurane or sevoflurane on postoperative quality of recovery after knee arthroscopy
Source: PLoS One. 2019 Aug 5;14(8):e0220733. doi: 10.1371/journal.pone.0220733 (PMC6681958; doi:10.1371/journal.pone.0220733)
Supplement: S1 Fig — Postoperative Quality of Recovery Scale. (PDF) [file pone.0220733.s003.pdf]

Pre- Anaesthetic Patient Questionnaire

Date Questionnaire conducted: ____ / ____ / ____ Days prior to the procedure: _____

NB: The physiological assessment should be conducted just prior to the procedure.

Physiological Factors	*Time to complete assessment: _____ seconds (transfer time to Test Summary)*
P1 Blood Pressure	Systolic: ____
P2 Heart Rate	HR: ____
P3 Temperature	Temp: ____ Method: ___________
P4 Respiration	Rate: ____
P5 SpO2	Value: ____ (Score 1-3)
P6 Airway	Value: ____ (Score 1-3)
P7 Agitation	Value: ____ (Score 1-3)
P8 Consciousness	Value: ____ (Score 1-3)
P9 Activity on command	Value: ____ (Score 1-3)
**Nociceptive Factors (Using Chart)**	*Time to complete assessment: _____ seconds (transfer time to Test Summary)*
N1 Pain level	Value: ____ (Score 1-5)
N2 PONV level	Value: ____ (Score 1-5)
**Emotional Factors (Using Chart)**	*Time to complete assessment: _____ seconds (transfer time to Test Summary)*
E1 Sadness / Depression	Value: ____ (Score 1-5)
E2 Nervousness / Anxiety	Value: ____ (Score 1-5)
**ADL Factors**	*Time to complete assessment: _____ seconds (transfer time to Test Summary)*
A1 Ability to stand without assistance	Value: ____ (Score 1-3)
A2 Ability to walk without assistance	Value: ____ (Score 1-3)
A3 Ability to eat or drink without assistance	Value: ____ (Score 1-3)
A4 Ability to dress without assistance	Value: ____ (Score 1-3)
**Cognitive Factors**	*Time to complete assessment: _____ seconds (transfer time to Test Summary)*
C1 Stating name, location & DOB	# correct responses: ____
C2 Numbers forward	# maximum correct rows: ____<br><br>1 **5, 6**<br>2 **1, 6, 4**<br>3 **7, 1, 9, 4**<br>4 **8, 3, 9, 6, 2**<br>5 **5, 2, 8, 7, 9, 4**<br>6 **6, 8, 5, 1, 3, 9, 7**
C3 Numbers backwards	# maximum correct rows: ____<br><br>1 **3, 4**<br>2 **1, 5 ,9**<br>3 **6, 2, 7, 3**<br>4 **8, 4, 7, 6, 1**<br>5 **9, 2, 4, 7, 1, 3**<br>6 **4, 1, 6, 9, 5, 2, 7**
C4 Word task	Number of words recalled: ____<br><br>DESK, RANGER, BIRD, SHOVEL, STOVE, MOUNTAIN, GLASSES, TOWEL, CLOUD, BOAT, LAMB, GUN, PENCIL, CHURCH, FISH
C5 Executive memory	Letter F # of words produced: ____

Patient refused to complete / continue with the study and withdrew: No (0), Yes (1)
Reason:………..
